# Advancements with Photobiomodulation in Post-Burn Management/Rehabilitation: A Comparative Study on Multiwave Locked System (MLS) LASER Therapy Outcomes

**DOI:** 10.3390/life16040611

**Published:** 2026-04-07

**Authors:** Ruxandra-Luciana Postoiu, Cristina Popescu, Silviu Marinescu, Gelu Onose

**Affiliations:** 1 Faculty of Medicine, University of Medicine and Pharmacy “Carol Davila”, 020021 Bucharest, Romania; silviu.marinescu@umfcd.ro (S.M.); gelu.onose@umfcd.ro (G.O.); 2 Neuromuscular Rehabilitation Clinic Division, Teaching Emergency Hospital “Bagdasar-Arseni”, 041915 Bucharest, Romania; 3 Plastic Surgery and Reconstructive Microsurgery Clinic Division, Teaching Emergency Hospital “Bagdasar-Arseni”, 041915 Bucharest, Romania

**Keywords:** burns, Multiwave Locked System (MLS) LASER, photobiomodulation, epithelialization, rehabilitation, function outcomes

## Abstract

**Background:** Severe burn injuries are associated with prolonged consequent wound healing, substantial symptoms burden, and delayed, sometimes incomplete, functional recovery. Photobiomodulation using Multiwave Locked System (MLS) LASER therapy has been proposed as an adjunctive intervention to support tissue repair and thereby improve rehabilitation outcomes, but related clinical evidence in burn populations remains limited. **Materials and Methods:** This comparative study included 65 patients with severe burn injuries, of whom 35 were prospectively treated with adjunctive MLS LASER therapy, in addition to standard care, and 30 retrospectively identified patients, who received standard care alone, served as controls. The primary outcome was the time until complete epithelialization, while secondary outcomes included: reduction in wound surface, pain intensity, pruritus severity, scar quality, and functional improvements. Assessments were performed at baseline and after a standardized follow-up period of up to 20 days. **Results**: Patients treated with MLS LASER therapy achieved complete epithelialization significantly earlier than controls (median 40 vs. 73 days, *p* < 0.001) and demonstrated greater wound area reduction (median 434 vs. 137 cm^2^, *p* = 0.0012). In multivariable analyses adjusted for burn extent, burn depth, age, and diabetes mellitus, considered as factors worsening evolution, MLS LASER therapy remained independently associated with shorter time to epithelialization and greater reduction in wound dimension. Significant improvements favoring the MLS group were also observed regarding pain, pruritus, scar quality, and functional outcomes, all assessed using specific evaluation tools (*p* < 0.001). **Conclusions:** Adjunctive MLS LASER therapy appears to be associated with improved wound healing dynamics and enhanced rehabilitation outcomes in patients with severe burn injuries. These findings should be interpreted with caution given the study limitations, including the non-randomized design and relatively small sample size. MLS LASER therapy may represent a promising adjunctive option in the conservative management of burn injuries; however, further prospective randomized studies are required to confirm these results and to define optimal treatment protocols.

## 1. Introduction

Burn injuries represent a major global public health challenge, being associated with substantial morbidity, long-term disability, and increased mortality worldwide [1]. According to the specialized literature and consistent with reports from the World Health Organization (WHO), more than 11 million individuals worldwide sustain burn injuries each year that are severe enough to require medical attention [1,2], and the associated mortality is estimated at approximately 180,000 deaths per year [1,3]. “Each rare disease affects fewer than 1 in 2000 people.” [4]. In comparison, severe burns exhibit a low population incidence, estimated at 0.2–2.9 cases per 10,000 inhabitants annually [5]. When expressed on the same population scale, this corresponds to approximately 0.04–0.58 cases per 2000 individuals, placing severe burns within epidemiologically rare occurrence ranges despite their traumatic etiology. The incidence is predominantly higher in countries with low or middle socio-economic status, where delayed access to medical care, the absence of specialized burn management centers, an increased prevalence of infections, and poor nutritional status are determining factors that amplify the risk of complications and increase burn-related mortality and/or complications and sequelae [6].

Epidemiological data highlight that the majority of patients who sustain burn injuries are predominantly young adults, most commonly between the second and fourth decades of life, with a male predominance of approximately 55–70% [7,8,9,10].

The etiology is dominated by thermal burns, particularly those caused by flames or fires, accounting for approximately 43% of cases, followed by scald injuries from hot liquids at around 34%, contact burns in approximately 9%, and electrical and chemical burns, each representing smaller proportions of about 4% and 3%, respectively [11].

In most regions worldwide, burn injuries occur predominantly in the domestic environment, although the exact proportion varies substantially depending on the socio-economic status of the population [12,13]. In low- and middle-income countries, numerous clinical and community-based studies indicate that approximately two-thirds to three-quarters of burn injuries occur in the home setting, and in some contexts, as many as 80–90% of reported burns are of domestic origin [1,14,15,16].

Studies from high-income countries report a different proportion, i.e., 50% to 70% of burn injuries in the domestic environment [5,17]. Burn injuries sustained in the workplace generally account for approximately 10–20% of cases, while the remaining injuries are associated with road traffic accidents or other circumstances [14,15].

Burns are defined as a complex type of trauma characterized by lesions to the skin and, in some cases, to underlying structures, resulting from exposure to thermal, electrical, chemical agents, or radiation, leading to protein denaturation, tissue necrosis, and impairment of local defense mechanisms [6]. Deep burn injuries trigger an inflammatory and metabolic cascade with both local and systemic manifestations, modulated by burn depth and the extent of the affected surface area, which renders burn management a major clinical challenge [3].

Burn severity is primarily determined by the depth of the injury and the percentage of total body surface area (TBSA) involved, as well as by associated factors such as patient age, comorbidities, and the presence of inhalation injuries [6].

Modern therapeutic management of post-burn pathology involves a staged, multidisciplinary approach that includes the following seven key components [6]: ranging from stabilization of vital functions [3] to the early initiation of a comprehensive rehabilitation program [18], including adequate fluid resuscitation [19], local wound care [20], infection prevention and control [21], as well as pain management [22].

### MLS LASER Therapy

In recent decades, photobiomodulation has emerged as an important modality in the management of tissue injuries due to its ability to modulate biological processes such as inflammation, microcirculation, and cellular regeneration [23], thus contributing to accelerated recovery, and interplay, functional enhancement, and consequent autonomy.

The term “LASER” originates from the English acronym Light Amplification by Stimulated Emission of Radiation [24,25], describing the physical principle through which light is amplified, followed with stimulated emission. The name was created by analogy with the “MASER” (Microwave Amplification by Stimulated Emission of Radiation), a precursor device, rather resembling the operating principle with microwave frequencies [26].

MLS LASER therapy represents an advanced photobiomodulation technology, a non-invasive therapeutic technique [23], that synchronously integrates the following two wavelengths: a continuous 808 nm pulsed beam, and a pulsed 905 nm emission [27].

The biological effects of MLS LASER therapy primarily derive from photon absorption at the mitochondrial cytochrome c oxidase, leading to increased ATP synthesis with stimulation of tissue repair processes [28]. Continuous emission contributes to the reduction in pro-inflammatory mediators such as IL-1β and TNF-α, decreasing edema and local inflammation [23]. Pulsed emission mainly exerts a neuro-modulatory effect, influencing peripheral nerve transmission and alleviating pain by reducing nociceptive hyperexcitability [29]. The synergistic action of the two components contributes to accelerated epithelialization, improved local perfusion, and stimulation of fibroblast activity—processes essential for wound healing, including in post-burn pathology [30,31].

Photobiomodulation effects are dose-dependent, with lower energy densities primarily inducing photochemical responses that promote tissue repair, while higher doses may lead to photothermal effects and potential tissue damage [32].

In this context, the present study aims to evaluate the impact of MLS LASER therapy on the epithelialization process and the clinical course of patients with severe burns, treated vs. non-treated with MLS LASER therapy.

## 2. Materials and Methods

The study was conducted in a Physical Rehabilitation Medicine Clinic Division of a Teaching Hospital, with the support of the Plastic Surgery and Reconstructive Microsurgery Clinic Division—which also has a functional unit for burn patients dedicated to acute burn care—in Bucharest, Romania, and encompassed a non-randomized comparative design.

All patients were initially evaluated in the Plastic Surgery Department, where therapeutic indications, including the need for surgical intervention (skin grafting), were established. The decision to perform or withhold grafting was made by the surgical team and individualized for each patient, based on burn depth, wound progression, overall clinical status, and patient consent. Subsequently, patients were referred to the Neuromuscular Rehabilitation Clinic for conservative management and adjunctive therapy, including MLS LASER.

Consequently, a total of 65 patients with burn injuries were included: 35 in the study group (53.8%) and 30 (46.2%) in the control group. The control group comprised patients retrospectively (between 2020 and 2021), and the patients in the study group, prospectively (since March 2023 in the present).

All patients received standard burn wound care in accordance with institutional clinical practice guidelines, including topical treatment with a compounded cream (zinc oxide—3 g, benzocaine—15 g, vitamin A—1 ampoule, vitamin E—1 ampoule, lanolin—15 g, and white petrolatum—85 g) along with appropriate dressings. In both groups, the topical agent was applied over the entire burn wound surface, followed by dressing application at 24 h intervals. In the MLS LASER cohort, LASER therapy sessions were performed prior to topical application and dressing, while the control group received only standard wound care.

### 2.1. Ethical Approval

The study was conducted in accordance with the Declaration of Helsinki and Good Clinical Practice (GCP) guidelines. Ethical approval was obtained from the Ethics Committee of the Teaching Emergency Clinical Hospital “Bagdasar-Arseni”, in Bucharest, under approval number 3180/10 February 2023. Written informed consent was obtained before study enrollment, following a detailed explanation of procedures, potential benefits, and risks. Participants were informed of their right to withdraw from the study at any time without affecting their standard care. All personal and clinical data were anonymized and coded to ensure confidentiality and were handled in strict compliance with applicable data protection regulations (GDPRs).

### 2.2. Objectives

The primary objective of this study was to assess the impact of MLS LASER therapy on time to epithelialization in patients with burn injuries.

Secondary objectives included evaluating patient tolerance, examining the relationship between LASER dosage and time to clinical improvement, and monitoring lesion progression using standardized assessment scales throughout the course of treatment.

### 2.3. Study Population and Data Acquisition

The study included patients from both cohorts whose burn injuries had occurred within the previous 30 days, in order to ensure comparability of healing stages. Data were collected prospectively for the MLS LASER group and retrospectively for the control group. All eligible patients meeting the inclusion criteria during the study period were included in order to reflect the real-world characteristics of the treated population.

Patient information was obtained from hospital medical records, including demographic characteristics (age, gender), burn characteristics (extent, location, and depth), comorbidities, and details of conventional and MLS LASER therapy. The retrospective data were obtained from hospital medical records and analyzed in anonymized form, in accordance with institutional regulations. Ethical approval was obtained prior to the initiation of the prospective phase of the study.

A standardized case-report form was used for all patients to ensure consistency in data collection (anonymized personal data, including my meta initials, are stored in Excel format available upon request).

Lesion measurements and assessment scores: Vancouver Scar Scale (VSS) [33], Visual Analog Scale (VAS) [34], 5-D Itch Scale [35], activities of daily living (ADLs) measured by the Katz Index [36], Barthel Index [37] and WHO Quality of Life-Bref (QOL) [38] were recorded at baseline and upon completion of the treatment protocol.

### 2.4. Inclusion and Exclusion Criteria

Inclusion criteria applied to the MLS LASER cohort: patients aged 18 years or older, clinically cooperative, and capable of actively participating in the treatment protocol, were eligible. All participants had confirmed burn injuries of grade IIA–IIB or III, were hemodynamically and respiratorily stable, and were willing to adhere to the treatment schedule and follow-up plan. Written informed consent was obtained prior to the study enrollment.

Exclusion criteria applied to the MLS LASER group: patients were excluded if they refused participation or were unable to provide informed consent. Additional exclusion criteria included: age under 18 years, unstable clinical status or acute exacerbation of chronic illnesses, active systemic or severe local, infection, active or recent history of malignancy, pregnancy or lactation, neurological disorders with high photosensitivity risk (e.g., active epilepsy, photosensitive migraine), severe psychiatric conditions limiting cooperation (e.g., dementia, psychosis, and severe cognitive impairment), known photosensitivity or adverse reactions to light-based therapies, presence of tattoos in the affected area, burns near the ocular region, or any other physical or psychological inability to comply with the treatment protocol and monitoring schedule.

For the retrospective control cohort, patients were selected from hospital records using equivalent criteria whenever possible, limited to those with complete baseline data and burn injuries of similar severity and duration (≤30 days from onset) to ensure comparability with the MLS LASER group.

### 2.5. Therapeutic Protocol/Treatment

Therapy sessions with MLS LASER were conducted within the Neuromuscular Rehabilitation Clinic Division. Patients who were unable to be mobilized received the therapy at bedside, whereas ambulatory patients were treated in a designated therapy room. During all procedures, both patients and medical staff wore protective gloves and goggles to prevent potential ocular injury. When other patients were present in the same room, a protective screen was installed to ensure eye safety and preserve patient privacy.

The therapeutic protocol was established in accordance with manufacturer recommendations and device-specific guidelines for MLS LASER therapy [39,40] with minor adjustments based on our own clinical experience and in alignment with the general principles of photobiomodulation.

LASER application was performed using the robotized scanning head of the MLS device (ASA LASER, Arcugnano (VI), Italy), which enables uniform energy distribution across the entire treatment area through automated movement patterns. The procedure was carried out in a non-contact manner, maintaining a distance of approximately 15–20 cm between the LASER source and the skin surface.

The MLS operates using synchronized dual wavelengths—808 nm continuous emission, associated with anti-inflammatory and anti-edematous effects, and 905 nm pulsed emission, associated with analgesic effects. The biostimulation treatment mode of the MLS device was applied. The target area was defined using a standard spot diameter of approximately 5 cm, and for larger lesions, the treated area was extended to ensure complete coverage of the affected surface.

A total of 10 consecutive treatment sessions were administered, with a frequency of one session every two days. The therapy was structured into two phases of five sessions each. In the first phase, LASER MLS parameters were set to 50% intensity (percentage of the device’s maximum output power), 700 Hz frequency, and a power density of 4 J/cm^2^, encompassing the entire burn lesion. In the second phase, the same treatment area and power density were maintained, while intensity was increased to 100% (maximum output power), frequency to 1000 Hz.

The duration of each session was not fixed but was automatically calculated by the device based on the treated surface area and the selected parameters. For a treatment area corresponding to the standard 5 cm spot diameter, session duration was approximately 4 min and 30 s in the first phase and approximately 2 min in the second phase. For larger lesions, treatment duration increased proportionally with the treated area.

The selected energy parameters (4 J/cm^2^) were defined based on manufacturer recommendations for the MLS LASER system (ASA) [39] and general principles of photobiomodulation therapy [28]. Lower energy densities were intentionally chosen to ensure safe application on burn tissue (which is well known to be very fragile in terms of trophicity and prone to an excessive inflammatory response) and to avoid potential thermal or, respectively, inhibitory effects, remaining within the recognized therapeutic window of photobiomodulation. This approach is consistent with current understanding of LASER–tissue interaction, where lower energy levels are associated with photochemical effects that promote tissue gentle repair, while higher energies may induce photothermal effects and increase the risk of tissue damage [23].

No preliminary dose-finding study was conducted. The protocol was applied according to device-specific guidelines within a clinically safe range. Considering that the use of MLS laser has successfully overcome the need for preliminary research steps, as cleared by the FDA in the USA and has European CE marking, our study is designed as a prospective clinical study with an applicative approach.

Patients were continuously monitored clinically throughout the sessions, with particular attention to signs of discomfort, excessive local warmth, or adverse skin reactions, with no thermal adverse effects observed. Additionally, burn lesions were assessed at baseline (upon admission) and at the end of the treatment period (up to 20 days), corresponding to the duration of the complete MLS LASER protocol (10 sessions), with the same evaluation time points applied in both groups to ensure comparability. Clinical-functional outcomes were further assessed using semi-quantitative/quantitative instruments, as previously described.

### 2.6. Statistical Analysis

Statistical analysis was performed using Statistical Package for the Social Sciences (SPSS) version 24, with Microsoft Excel 2021 used for data management and figure preparation. Continuous variables were evaluated for distribution characteristics through graphical inspection methods (including box-plot visualization) and descriptive statistical indicators. Assessment of data distribution indicated skewness and the presence of outliers for several outcome measures. Therefore, continuous variables are reported as median and interquartile range (IQR), whereas categorical variables are reported as counts and percentages.

Between-group comparisons of continuous variables were performed using the Mann–Whitney U test. This non-parametric approach was selected in accordance with distributional characteristics and sample size considerations. Comparisons of categorical variables were conducted using the Chi-square test, while Fisher’s exact test was applied when expected cell counts were <5. For continuous variables approximating normal distribution, between-group comparisons were performed using Welch’s t-test, which accounts for unequal variances between groups. Changes in clinical, patient-reported, and functional outcomes were calculated as differences between baseline and follow-up measurements (Δ values) and were compared between groups using non-parametric methods.

To identify factors independently associated with time to complete epithelialization and wound area reduction, multivariable linear regression analyses were performed. Variables included in the adjusted models were selected based on clinical relevance and baseline imbalance and comprised treatment group (MLS vs. control), burn extent (percentage of total body surface, %TBSA), burn depth, diabetes mellitus, and age (included in sensitivity analysis). Regression results are reported as unstandardized coefficients (β) with corresponding 95% confidence intervals (CIs). Model performance was evaluated using the coefficient of determination (adjusted R^2^).

All statistical tests were two-tailed, and a *p*-value < 0.05 was considered statistically significant. A 95% confidence level was applied for all inferential analyses.

## 3. Results

### 3.1. Study Population and Baseline Characteristics

The distribution between the two cohort groups, the study one (35 patients = 53.8%) and the control group (30 patients = 46.2%), were not equal.

Male patients predominated in both study groups. In the MLS-treated cohort, 25 patients (71.4%) were male, and 10 (28.6%) were female, while in the control group, 21 patients (70.0%) were male and nine (30.0%) were female, with no significant difference in gender distribution (Chi-square test, χ^2^ = 0.015, *p* = 0.90).

The mean age of patients in the MLS-treated group was 44.9 ± 15.1 years, whereas patients in the control group had a mean age of 50.7 ± 14.2 years. Although patients in the control group tended to be older, the difference between groups did not reach statistical significance (Welch’s t = −1.58, *p* = 0.12).

Regarding the place of residence, most patients in both groups originated from urban areas. In the MLS-treated group, 26 patients (74.3%) were from urban environments and nine (25.7%) from rural areas, whereas in the control group, 19 patients (63.3%) lived in urban settings and 11 (36.7%) in rural settings. Statistical comparison did not identify a significant difference concerning residence distribution between the two groups (Chi-square test, χ^2^ = 0.91, *p* = 0.34).

Smoking status was also comparable between cohorts: in the MLS-treated group, 22 patients (62.9%) were active smokers, compared to 14 patients (46.7%) in the control group. Although a higher proportion of smokers was observed among patients receiving MLS therapy, the difference between groups did not reach statistical significance (Chi-square test, χ^2^ = 1.72, *p* = 0.19).

The presence of associated comorbidities was assessed by identifying chronic conditions with potential impact on the wound healing process. Diabetes mellitus was significantly more prevalent in the control group, being present in 12 patients (40.0%), compared to five patients (14.3%) in the MLS-treated group, a difference that reached statistical significance (Chi-square test, χ^2^ = 5.32, *p* = 0.021). Given this imbalance, diabetes mellitus was considered a potential confounding factor and was therefore included as a covariate in subsequent multivariable analyses. In contrast, other relevant comorbidities, including arterial hypertension and obesity, were identified in 31.4% of patients in the MLS group and in 43.3% of patients in the control one, without statistically significant differences (Chi-square test, χ^2^ = 0.97, *p* = 0.32).

Baseline demographic and clinical characteristics of the study population are summarized in Table 1.

### 3.2. Burns Characteristics

The extent of a burn injury, expressed as a percentage of total body surface area (%TBSA), was analyzed. The median TBSA was 14% (IQR 10–22) in the MLS-treated group and 16.5% (IQR 12–22) in the control one, with no significant difference between these cohorts (Mann–Whitney U = 454, *p* = 0.41).

Regarding burn depth, mixed partial-thickness injuries predominated in both groups. In the MLS-treated cohort, grade IIA burns were identified in five patients (14.3%), grade IIA–IIB burns in 15 patients (42.9%), grade IIB burns in seven patients (20.0%), and mixed IIB–III injuries in seven patients (20.0%); a single case of full-thickness burn (grade III) was recorded (2.8%). A similar distribution was observed in the control group, with no statistically significant difference regarding burn depth categories between the cohorts (Chi-square test, χ^2^ = 4.21, *p* = 0.38).

Thermal burns accounted for the vast majority of injuries in both groups. In the MLS-treated cohort, 34 patients (97.1%) sustained thermal injuries, while a single case (2.9%) was attributed to an electrical mechanism. Similarly, in the control group, thermal burns were observed in 29 patients (96.7%), with only one patient (3.3%) presenting with an electrical one. Comparative analysis using the Chi-square test did not reveal a statistically significant difference in the distribution of burn types between the two groups (χ^2^ ≈ 0.01, *p* = 0.92).

Burn injuries were most commonly caused by exposure to hot liquids in both groups. In the MLS-treated cohort, hot-liquid burns were observed in 42.9% of patients, flame-related injuries in 28.6%, and explosion-associated burns in 25.7%. A comparable pattern was observed in the control group, with hot liquids responsible for 43.3% of injuries, followed by flame exposure at 30.0% and explosion-related mechanisms at 26.7%. Statistical comparison using the Chi-square test demonstrated no significant differences (χ^2^ = 0.04, *p* = 0.99).

The anatomical distribution of burns was as follows: in the MLS-treated cohort, upper limb involvement was observed in 54.3% of patients, lower limb in 62.9%, and trunk in 31.4%. Similarly, in the control group, burns affected the upper limbs in 53.3% of cases, the lower limbs in 60.0%, and the trunk in 33.3%. No statistically significant differences were observed between groups (upper limb: χ^2^ = 0.01, *p* = 0.92), (lower limb: χ^2^ = 0.05, *p* = 0.82), or (trunk χ^2^ = 0.03, *p* = 0.86), based on the Chi-square test.

Surgical skin grafting was required in a single patient in the MLS-treated group and in none of the control patients; this difference was not statistically significant (Fisher’s exact test, *p* = 1.00).

Evaluated parameters and the statistical differences are presented below, in (Table 2).

### 3.3. Primary Outcome: Time to Complete Epithelialization

**Figure 1:**
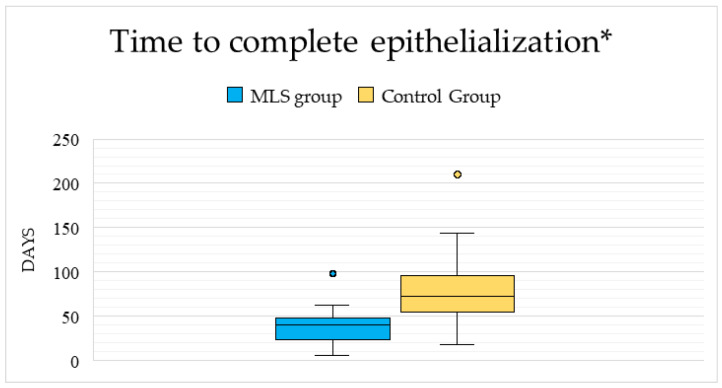
Time to complete epithelialization in the MLS-treated and in the control, groups. * box plots represent median values, interquartile ranges, and extreme values.

Time to complete epithelialization was defined as the number of days between the date of burn injury and the date of documented complete epithelial closure. Patients in the MLS-treated group achieved complete epithelialization significantly earlier, with a median time of 40 days (IQR 24–47.5), compared to 73 days (IQR 54.5–95.8) in the control group, and this difference was statistically significant (Mann–Whitney U = 155.5, *p* < 0.001). These differences are illustrated in Figure 1.

### 3.4. Multivariable and Sensitivity Analyses for Time to Complete Epithelialization

The results of the multivariable linear regression analysis (independent associations of MLS LASER therapy, TBSA, burn depth, and diabetes mellitus with time to epithelialization—Table 3) indicated that the first two were significant independent predictors. MLS treatment was correlated with a reduction in healing time of 38.9 days (95% CI: −50.6 to −27.2; *p* < 0.001), whereas each 1% increase in TBSA was related to delayed epithelialization, with an estimated increase of 2.14 days (95% CI: 0.96 to 3.31; *p* < 0.001). Although burn depth and diabetes mellitus were associated with a delay of the healing process, 3.91 and 2.99 days, respectively, neither variable remained a statistically significant independent predictor in the adjusted model (*p* = 0.286 and *p* = 0.665). Overall, the model explained 62.2% of the variance in time to epithelialization (adjusted R^2^ = 0.622*), * model performance: adjusted R^2^ = 0.622.

In sensitivity analysis with additional adjustment for age, the association between MLS LASER therapy and shorter time to complete epithelialization remained consistent and statistically significant (β = −38.1 days; *p* < 0.001). The effect of TBSA was preserved, while age showed a borderline association with epithelialization time (*p* = 0.062). Diabetes mellitus and burn depth remained non-significant predictors.

### 3.5. Wound Surface Reduction

**Figure 2:**
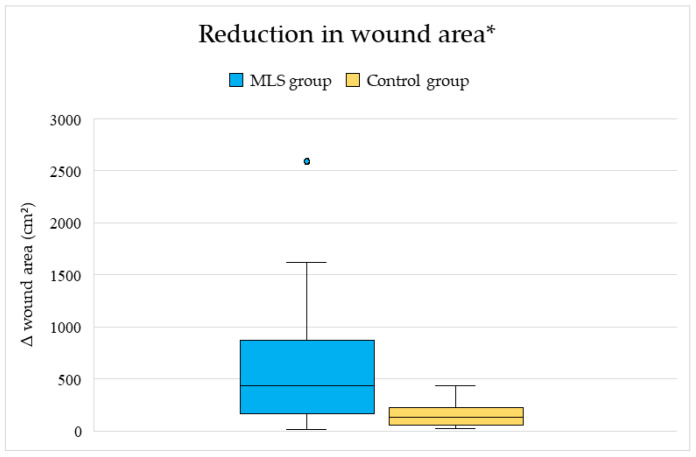
Reduction in wound area* (Δ cm^2^) in the MLS-treated and control groups. * box plots represent median values, interquartile ranges, and extreme values.

In the MLS-treated group, the median wound area decreased from 448 cm^2^ (IQR 166.5–918) at baseline to 0 cm^2^ (IQR 0–5) at follow-up, corresponding to a median reduction of 434 cm^2^ (IQR 166.5–870). In contrast, in the control group, the median wound area decreased from 336 cm^2^ (IQR 115.5–673.8) to 166.5 cm^2^ (IQR 48.5–296.3), with a median reduction of 137 cm^2^ (IQR 57.3–241.5). The between-group difference in wound area reduction was statistically significant (Mann–Whitney U = 772.5, *p* = 0.0012) and is illustrated in Figure 2.

**Figure 3:**
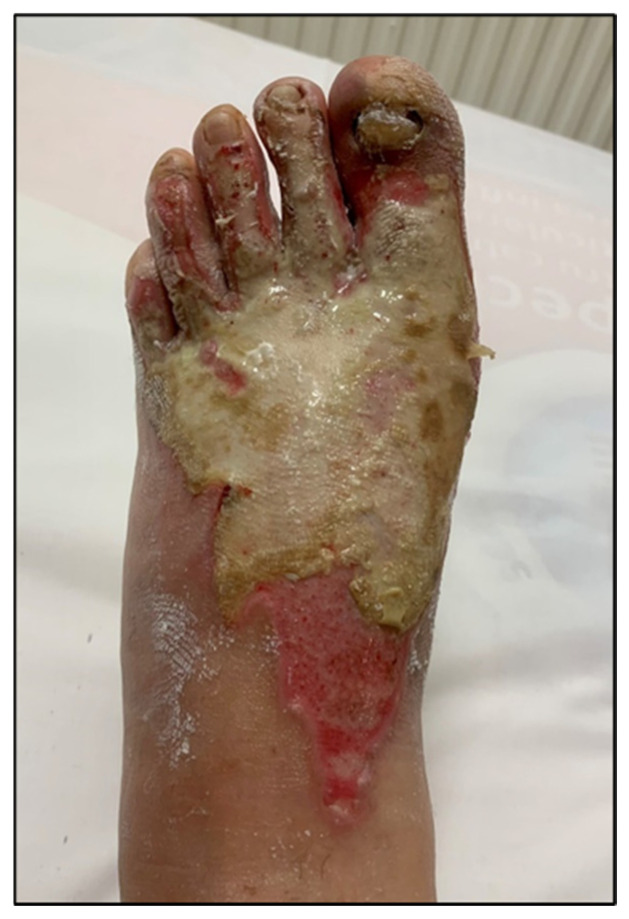
Post-combustional lesion on the dorsal surface of the foot, before initiating MLS LASER therapy.

**Figure 4:**
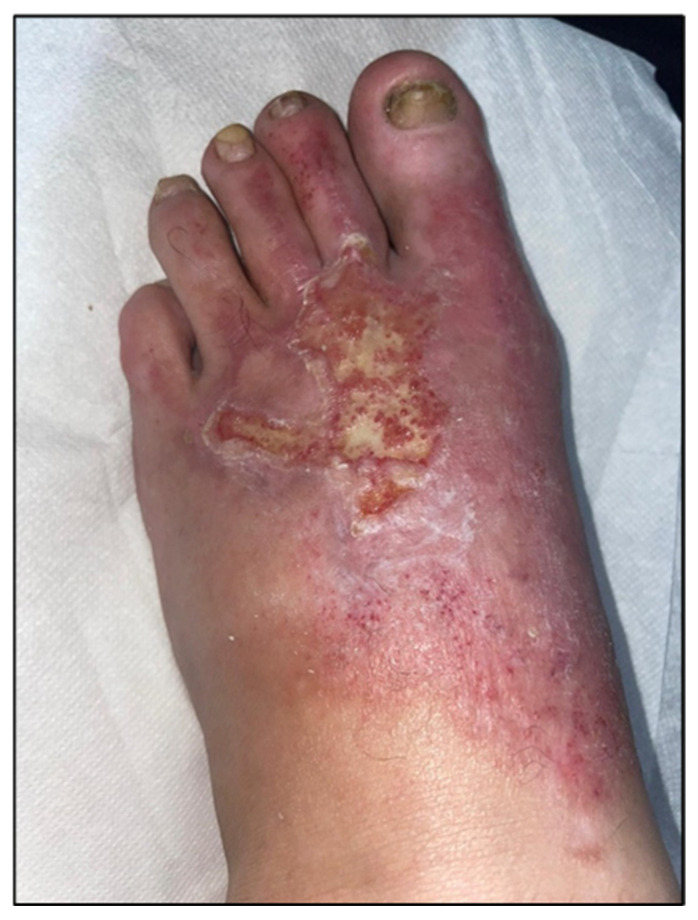
Post-combustional lesion on the dorsal surface of the foot, after the 10 sessions of MLS LASER therapy.

**Figure 5:**
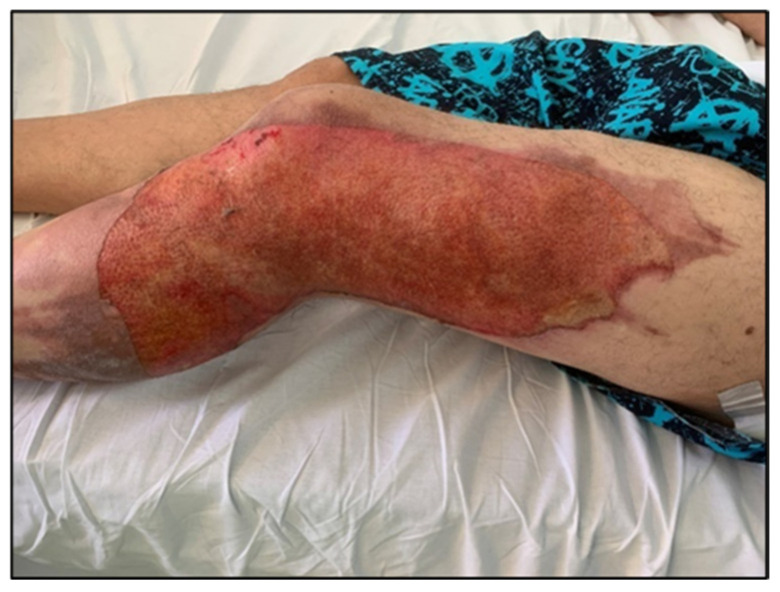
Post-burn lesion on the lateral surface of the lower limb, before initiating MLS LASER therapy.

**Figure 6:**
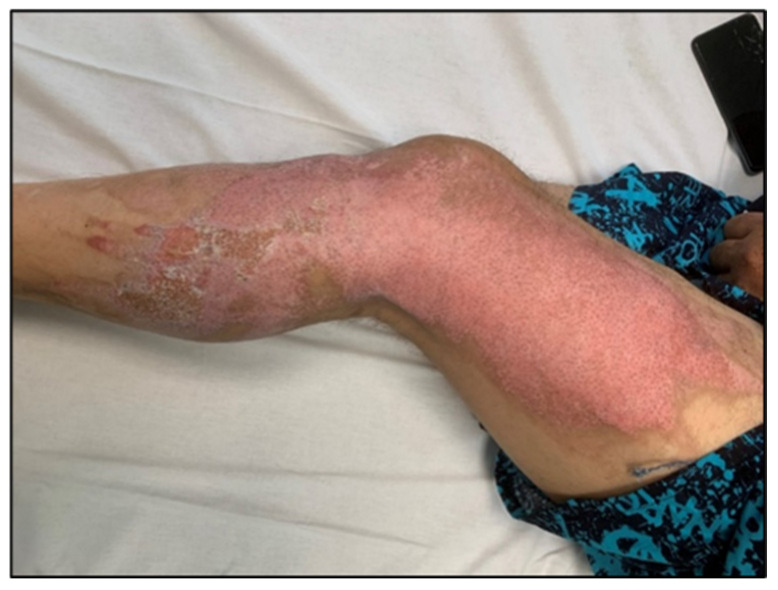
Post-burn lesion on the lateral surface of the lower limb, after the 10 sessions of MLS LASER therapy.

**Figure 7:**
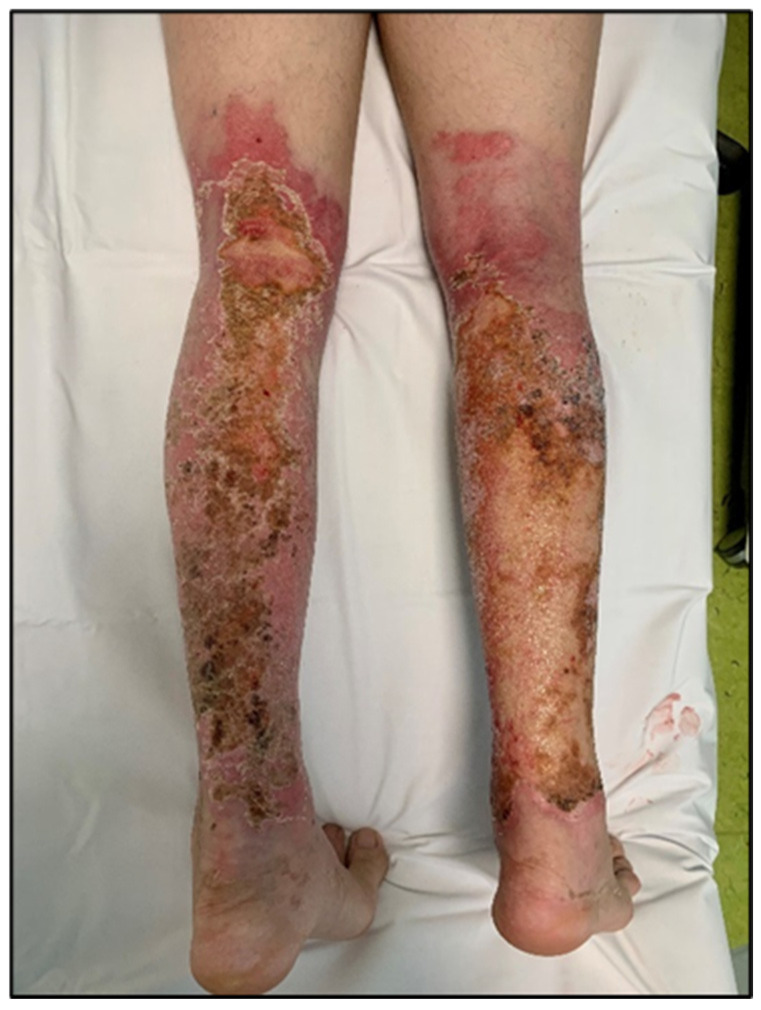
Post-combustional lesion on the posterior face of the lower limbs, before initiating MLS LASER therapy.

**Figure 8:**
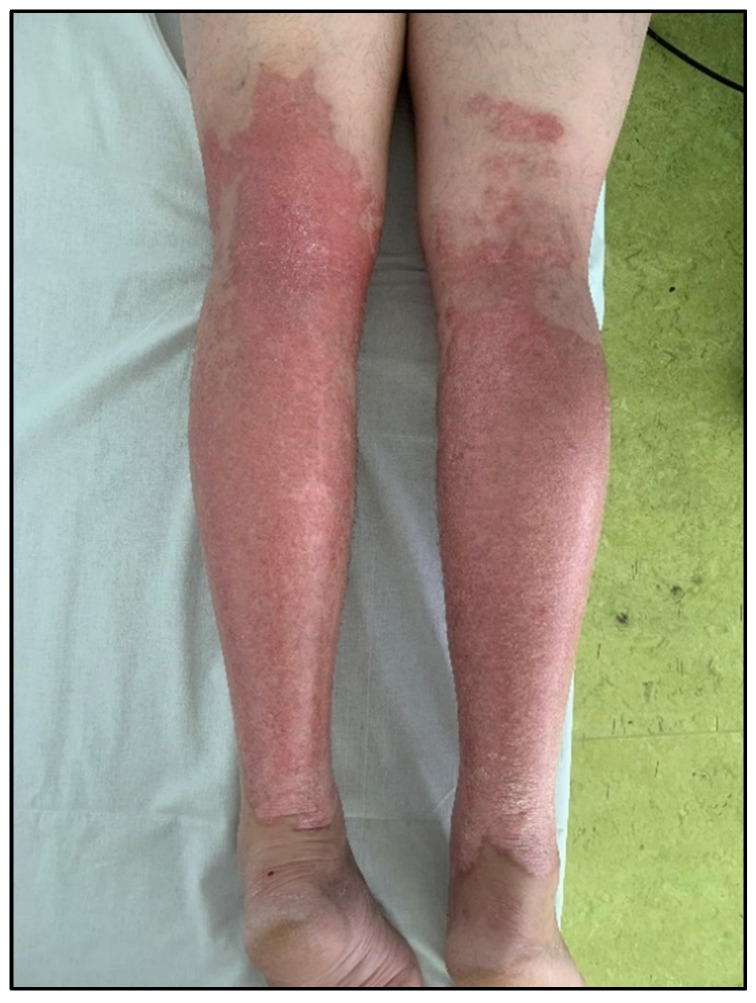
Post-combustional lesion on the posterior face of the lower limbs, after the MLS LASER therapy.

For enhanced transparency, the present paper includes a few selected images from the MLS cohort, obtained during the study, as shown in Figure 3, Figure 4, Figure 5, Figure 6, Figure 7 and Figure 8 (describing the post-burn lesion before starting MLS laser therapy and the final result after the sessions of MLS laser, according to the therapeutic protocol described in Section 2).

In multivariable linear regression analysis, MLS LASER therapy was independently associated with a significantly greater reduction in wound area after adjustment for burn extent (%TBSA), burn depth, and diabetes mellitus (Table 4). MLS therapy was associated with an additional reduction of 425.5 cm^2^ in wound surface (Δ cm^2^), compared to standard care (95% CI 226.7–624.2; *p* < 0.001). TBSA was also independently associated with wound area decrease (β = 21.25 cm^2^ per 1% TBSA; *p* < 0.001). Burn depth showed a modest but significant association with wound area diminution, while diabetes mellitus was not a significant predictor in the adjusted model. Overall, the model explained 36.6% of the variance in wound area reduction (adjusted R^2^ = 0.366*), * model performance: adjusted R^2^ = 0.366.

### 3.6. Patient-Reported and Functional Outcomes

Pain intensity, assessed using VAS [34], showed a marked reduction over the follow-up period in patients receiving adjunctive MLS LASER therapy. Median VAS scores decreased from 8 (IQR 7–10) at baseline to 0 (IQR 0–2) at follow-up, corresponding to a median pain reduction of eight points (IQR 7–8). By comparison, patients managed with standard care alone experienced a more modest decrease in pain severity, with median VAS scores declining from 9 (IQR 8–10) to 6 (IQR 5–7), yielding a median reduction of three points (IQR 2–3). The difference in pain reduction between cohorts was statistically significant (Mann–Whitney U = 766.0, *p* < 0.001).

Scar quality, evaluated using VSS [33], also improved to a greater extent among patients treated with MLS therapy. Median VSS scores decreased from 9 (IQR 8–11) at baseline to 3 (IQR 2–4) at follow-up, corresponding to a median improvement of five points (IQR 5–7). In contrast, the comparator cohort demonstrated a smaller improvement, with median scores decreasing from 9 (IQR 7.25–9) to 7.5 (IQR 5.25–8), resulting in a median improvement of two points (IQR 1–2). The between-group difference in scar quality improvement was statistically significant (Mann–Whitney U = 1019.0, *p* < 0.001).

Results according to pruritus severity, measured using the 5-D Itch Scale, are as follows: [35] median scores declined from 20 (IQR 15.5–21) at baseline to 5 (IQR 5–6) at follow-up, corresponding to a median reduction of 13 points (IQR 10–15), among the MLS group. In contrast, the standard-care group exhibited a reduction from 20 (IQR 19–20) to 11 (IQR 9–12), with a median improvement of nine points (IQR 8–10). The difference in pruritus reduction between cohorts was statistically significant (Mann–Whitney U = 813.0, *p* < 0.001).

Considering independence in activities of daily living, patients in the MLS cohort demonstrated an increase in median ADL scores from 3 (IQR 2–5) at baseline to 6 (IQR 5–6) at follow-up, corresponding to a median improvement of two points (IQR 1–3). In the comparator group, median ADL scores slightly increased from 4 (IQR 3–4) to 4 (IQR 4–5), with a median improvement of one point (IQR 0–1). The between-group difference in ADL improvement was statistically significant (Mann–Whitney U = 808.5, *p* < 0.001).

Functional independence, assessed using the Barthel Index [37], followed a similar pattern. Median Barthel scores increased from 70 (IQR 55–80) at baseline to 100 (IQR 90–100) at follow-up, corresponding to a median functional gain of 25 points (IQR 17.5–30). By contrast, patients receiving conventional treatment alone showed an increase from 57.5 (IQR 50–68.8) to 70 (IQR 65–83.8), with a median improvement of 10 points (IQR 6.3–15). This difference was statistically significant (Mann–Whitney U = 165.0, *p* < 0.001).

From the perspective of quality of life [38], in the MLS-cohort, the median WHOQOL-BREF score increased from 41 points (IQR 32.5–47.5) at baseline to 103 points (IQR 97.5–108.5) at follow-up, corresponding to a median gain of 63 points (IQR 55.5–71.0), whereas in the control cohort scores rose from 44.5 (IQR 38.0–48.75) to 79 points (IQR 72.25–84), corresponding to a median improvement of 35.5 points (IQR 28.0–43.75). Both cohorts difference demonstrated a statistically significant improvement, with the mention that the MLS-treated group showed a more substantial gain than the other group (Mann–Whitney U = 968.5, *p* < 0.001).

Across all patient-reported and functional outcomes, results consistently favored the MLS-treated group, as summarized in Table 5.

## 4. Discussion

The findings of the present study indicate that adjunctive MLS LASER therapy is associated with accelerated wound healing, improved symptom control, and enhanced functional recovery in patients with severe burn injuries. Compared to the conventional care group, the cohort treated with MLS LASER therapy was accompanied by a shorter time to complete epithelialization (median 40 vs. 73 days, *p* < 0.001), along with a greater reduction in wound surface (median Δ 434 cm^2^ vs. 137 cm^2^, *p* = 0.0012). These structural improvements were accompanied by a clinically meaningful reduction in pain intensity, pruritus severity, better scar quality, and enhanced independence in ADLs.

The accelerated epithelialization observed aligns with preclinical and clinical evidence on photobiomodulation. Low-level laser therapy has been shown, in animal and cell studies, to stimulate fibroblast proliferation, enhance angiogenesis, and modulate local inflammatory responses, thereby promoting tissue repair [23,41]. The observed median reduction of 40 days in epithelialization is particularly relevant, as delayed wound closure is associated with prolonged hospitalization, increased risk of wound infection, hypertrophic scar formation, and delayed functional recovery. Previous clinical studies have demonstrated that extended time to wound closure negatively affects both rehabilitation timelines and long-term functional outcomes in burn populations [42,43].

Specifically, to our knowledge, this is one of the first clinical studies evaluating MLS LASER therapy as a photobiomodulation intervention in burn patients. Current evidence regarding its use in burn wound management remains limited, with only isolated reports available, including single-case descriptions [44]. It should be noted that MLS LASER exceeds the therapeutic capabilities of classical laser devices, for which previous studies in burn treatment do exist; however, we emphasize and underline that MLS LASER represents an advanced device that is both conceptually and technically different, representing a next level in photobiomodulation. Although direct comparison is not feasible due to the scarcity of data, the findings of the present study appear to be consistent with these preliminary observations, suggesting a potential beneficial effect of MLS LASER therapy.

Scar quality was also significantly improved in MLS-treated patients than in controls (*p* < 0.001). While standardized clinical data on laser-based scar modulation in burn populations remain limited, evidence from non-burn surgical and traumatic wounds suggests that photobiomodulation can influence collagen synthesis and fibroblast activity, thereby contributing to improved collagen organization and tissue remodeling [45]. Our findings are in line with these observations, suggesting that adjunctive MLS therapy may contribute not only to superficial epithelial closure but also to deeper tissue remodeling, which is critical for long-term functional outcomes.

Functional recovery represents a key endpoint in burn rehabilitation. In this study, functional independence and ADLs, evaluated using the Barthel Index and Katz ADL scale, showed significantly greater improvement in the MLS group compared with standard care alone (both *p* < 0.001). This improvement likely reflects a combination of faster wound healing, reduced pain, and decreased pruritus, enabling earlier mobilization and active participation in rehabilitation programs, including those aimed at improving mobility. While direct evidence linking laser therapy to functional outcomes in burn populations remains limited, studies in other post-surgical and traumatic contexts have demonstrated similar benefits [46]. These results are consistent with the potential of MLS LASER therapy as a component of comprehensive, multidisciplinary burn rehabilitation strategies.

An important strength of this study lies in its multidimensional approach to outcome assessment, combining validated evaluation instruments with an integrated analysis of structural, symptomatic, and functional parameters, alongside rigorous statistical adjustment for key predictors such as burn size, depth, and comorbidities.

Several limitations should be acknowledged.

The non-randomized study design and rather modest (considering severe burns as being rare conditions) sample size limit the generalizability of the findings. Furthermore, the heterogeneity of LASER protocols reported in the literature, together with the well-described dose-dependent and biphasic biological response to photobiomodulation, underscores the need for standardized treatment parameters [28]. Although the treatment protocol applied in the present study was associated with favorable clinical outcomes, further research is required to confirm these findings. Future research should prioritize multicenter, randomized controlled trials with larger cohorts, strengthened standardization of MLS LASER therapy protocols, extended follow-up periods, and comprehensive evaluation of long-term burn wound healing, scar maturation, and functional recovery.

The relatively prolonged time to complete epithelialization observed in our cohort, with a median of 40 days in the MLS-treated group and 73 days in the control group, should be interpreted in the context of a predominantly conservative therapeutic approach, without early surgical intervention (skin grafting). Under these conditions, wound closure may have involved combined healing mechanisms, including both epithelialization and contraction processes [47], which may influence the comparability of our results with studies based on early surgical management. Nevertheless, despite the longer time to epithelialization, patients treated with MLS LASER therapy demonstrated a significantly greater improvement in scar quality, as assessed using the Vancouver Scar Scale (VSS), with a median improvement of five points, compared with two points in the control group (*p* < 0.001), suggesting a potential beneficial effect of the therapy on tissue remodeling.

Pain and pruritus are closely related to the stage of wound healing, being influenced by inflammatory processes and the degree of epithelialization, as well documented in the literature [48]. In our study, assessments using VAS and the 5-D Itch Scale were performed within a standardized time frame (up to 20 days), independent of complete epithelialization. At the final evaluation, some wounds in both groups were not fully epithelialized, more frequently in the control group, which may have influenced symptom scores. Therefore, these findings should be interpreted with caution.

Diabetes mellitus, a known factor affecting wound healing [49], was more prevalent in the control group. Although adjusted analyses did not show a statistically significant independent effect, the relatively small number of diabetic patients may have limited statistical power, and a type II error cannot be excluded.

Additional limitations include the use of a retrospective control group, as well as the absence of a direct comparison between MLS and classical (monowave) LASER systems, which would have allowed a more direct evaluation of MLS-specific effects. This comparison was not feasible within the current study due to the unavailability of alternative LASER devices in our clinical setting. Consequently, this limits the ability to clearly differentiate MLS-specific effects from those of conventional photobiomodulation approaches. Future studies should therefore include comparative designs involving three groups: standard care without photobiomodulation, standard care with classical photobiomodulation, and standard care with MLS LASER therapy, in order to better define the specific contribution and potential advantages of this technology.

From a clinical perspective, the magnitude and consistency of the observed effects across multiple domains suggest a potentially meaningful impact of MLS LASER therapy on the overall rehabilitation trajectory of burn patients, supporting its consideration as an adjunctive intervention within standard burn care.

## 5. Conclusions

This study suggests that adjunctive MLS LASER therapy may enhance the healing and rehabilitation process in patients with severe burn injuries when added to standard care. Across multiple clinically relevant domains, MLS LASER therapy was associated with faster wound closure, greater reduction in wound surface, and improved patient-reported and functional outcomes.

The observed improvements in pain, pruritus, scar quality, and independence in daily activities highlight the potential of MLS LASER therapy to facilitate a more efficient rehabilitation trajectory. However, these findings should be interpreted in the context of the healing trajectory, including the relatively prolonged epithelialization time and the predominantly conservative therapeutic approach, in which patients included in this study were not considered suitable candidates for early surgical intervention (skin grafting). Under these conditions, wound closure may involve combined mechanisms such as epithelialization and contraction, which may limit direct comparability with studies based on early surgical management.

Importantly, the association between MLS LASER therapy and improved outcomes remained consistent after accounting for major clinical factors related to burns severity, including their extent, depth, and relevant comorbidities—such as diabetes mellitus, in some of the patients. Although diabetes mellitus is a known factor that may delay wound healing and was more prevalent in the control group, the adjusted analysis did not show a statistically significant independent effect, and this finding should be interpreted with caution.

Furthermore, the results should be considered in light of the study limitations, including the non-randomized design, the use of a retrospective control group, and the relatively small sample size, which may affect the generalizability of the findings.

While these findings indicate a promising role for MLS LASER therapy as an adjunctive intervention in burns care, they should be interpreted with caution. Further prospective randomized studies are required to confirm its effectiveness, define optimal treatment protocols, and evaluate long-term clinical and functional outcomes.

Overall, MLS LASER therapy appears to be an efficient adjunctive intervention in multidisciplinary burn management, with real potential to enhance both healing efficiency—including a reduction in time to epithelization—and the speed of wound closure, and rehabilitation functional outcomes in patients with severe post-combustional lesions.

## Figures and Tables

**Table 1:** Baseline demographic and clinical characteristics of the study population.

Characteristic	MLS Group (n = 35)	Control Group (n = 30)	*p*-Value
Age, years	44.9 ± 15.1	50.7 ± 14.2	0.12 ^a^
Gender, n (%)			0.90 ^b^
Male	25 (71.4%)	21 (70.0%)	
Female	10 (28.6%)	9 (30.0%)	
Residence, n (%)			0.34 ^b^
Urban	26 (74.3%)	19 (63.3%)	
Rural	9 (25.7%)	11 (36.7%)	
Smoking status, n (%)			0.19 ^b^
Smoker	22 (62.9%)	14 (46.7%)	
Non-smoker	13 (37.1%)	16 (53.3%)	
Diabetes mellitus, n (%)			0.021 ^b^
Yes	5 (14.3%)	12 (40.0%)	
No	30 (85.7%)	18 (60.0%)	
Other comorbidities *, n (%)	11 (31.4%)	13 (43.3%)	0.32 ^b^

Data are presented as mean: ±standard deviation or number (percentage), as appropriate. ^a^ Welch’s t-test. ^b^ Chi-square test. * other comorbidities include arterial hypertension and obesity.

**Table 2:** Burns characteristics of the patients included in our research.

Characteristic	MLS Group (n = 35)	Control Group (n = 30)	*p*-Value
TBSA (%), median (IQR)	14% (10–22)	16.5% (12–22)	0.41 ^a^
Burn depth, n (%)			0.38 ^b^
IIA	5 (14.3%)	3 (10.0%)	
IIA-IIB	15 (42.9%)	13 (43.3%)	
IIB	7 (20.0%)	10 (33.3%)	
IIB-III	7 (20.0%)	4 (13.3%)	
III	1 (2.8%)	0 (0%)	
Burn type, n (%)			0.92 ^b^
Thermal	34 (97.1%)	29 (96.7%)	
Electrical	1 (2.9%)	1 (3.3%)	
Burn mechanism, n (%)			0.99 ^b^
Hot liquids	15 (42.9%)	13 (43.3%)	
Flame	10 (28.6%)	9 (30.0%)	
Explosion	9 (25.7%)	8 (26.7%)	
Burn localization, n (%) *			
Upper limbs	19 (54.3%)	16 (53.3%)	0.92 ^b^
Lower limbs	22 (62.9%)	18 (60.0%)	0.82 ^b^
Trunk	11 (31.4%)	10 (33.3%)	0.86 ^b^
Skin grafting, n (%)	1 (2.9%)	0 (0%)	1.00 ^c^

^a^ Mann–Whitney U test. ^b^ Chi-square test. ^c^ Fisher’s exact test. * percentages may exceed 100% due to multiple burn locations per patient.

**Table 3:** Multivariable linear regression analysis of factors associated with time to complete epithelialization.

Variable	β (days)	95% CI	*p*-Value
MLS treatment	−38.89	−50.56 to −27.22	<0.001
TBSA (%)	+2.14	0.96 to 3.31	<0.001
Burn depth	+3.91	−3.28 to 11.10	0.286
Diabetes mellitus	+2.99	−10.52 to 16.49	0.665

Regression coefficients are reported as unstandardized β values. Burn depth was modeled as an ordinal variable (IIA to III).

**Table 4:** Multivariable linear regression analysis of factors associated with wound area reduction (Δ cm^2^).

Variable	β (cm^2^)	95% CI	*p*-Value
MLS treatment	+425.5	226.7 to 624.2	<0.001
TBSA (% per 1% increase)	+21.25	9.95 to 32.55	<0.001
Burn depth (per grade increase)	+118.2	5.8 to 230.6	0.040
Diabetes mellitus	+140.6	−99.2 to 380.4	0.245

Regression coefficients are reported as unstandardized β values. Burn depth was modeled as an ordinal variable. TBSA = total body surface area.

**Table 5:** Comparison of patient-reported and functional outcomes between the MLS-treated and control groups.

Outcome	MLS Group (n = 35)	Control Group (n = 30)	*p*-Value
Pain (VAS)	8 (7–8)	3 (2–3)	<0.001
Scar quality (VSS)	5 (5–7)	2 (1–2)	<0.001
Pruritus (5-D Itch)	13 (10–15)	9 (8–10)	<0.001
Independence in activities of daily living (ADLs)	2 (1–3)	1 (0–1)	<0.001
Functional independence (Barthel)	25 (17.5–30)	10 (6.3–15)	<0.001
Quality of Life (WHOQOL-BREF)	63 (55.5–71)	35.5 (28–43.75)	<0.001

## Data Availability

The data are not publicly available due to privacy and ethical restrictions but are available from the corresponding author upon reasonable request.

## Abbreviations

The following abbreviations are used in this manuscript:

MLS	Multiwave Locked System
WHO	World Health Organization
TBSA	Total Body Surface Area
VAS	Visual Analog Scale
ADLs	Activities of Daily Living
WHOQOL-BREF	World Health Organization Quality of Life-BREF
IQR	Interquartile Range
CIs	Confidence Intervals

## References

[1] World Health Organization Burns—Key Facts. https://www.who.int/news-room/fact-sheets/detail/burns.

[2] Radzikowska-Büchner E., Łopuszyńska I., Flieger W., Tobiasz M., Maciejewski R., Flieger J. (2023). An Overview of Recent Developments in the Management of Burn Injuries. Int. J. Mol. Sci..

[3] Jeschke M.G., van Baar M.E., Choudhry M.A., Chung K.K., Gibran N.S., Logsetty S. (2020). Burn injury. Nat. Rev. Dis. Primers.

[4] What Is a Rare Disease?. https://www.eurordis.org/information-support/what-is-a-rare-disease/.

[5] Brusselaers N., Monstrey S., Vogelaers D., Hoste E., Blot S. (2010). Severe burn injury in Europe: A systematic review of the incidence, etiology, morbidity and mortality. Crit. Care.

[6] Herndon D.N. (2018). Total Burn Care.

[7] Alipour J., Mehdipour Y., Karimi A. (2020). Epidemiology and outcome analysis of 3030 burn patients with an ICD-10 approach. Ann. Burn. Fire Disasters.

[8] Tian H., Wang L., Xie W., Shen C., Guo G., Liu J., Han C., Ren L., Liang Y., Tang Y. (2018). Epidemiologic and clinical characteristics of severe burn patients: Results of a retrospective multicenter study in China, 2011–2015. Burn Trauma.

[9] Stojanović M., Marinković M., Jurišić M., Miličić B., Stojičić M., Jovanović M., Jeremić J., Dimić N., Srećković S., Drača Cetušić I. (2025). Epidemiological characteristics of hospitalized burn patients—A 10-year retrospective study in a major burn center in Serbia. Life.

[10] Pieptu V., Moscalu R., Mihai A., Moscalu M., Pieptu D., Azoicăi D. (2022). Epidemiology of hospitalized burns in Romania: A 10-year study on 92,333 patients. Burns.

[11] Schaefer T.J., Szymanski K.D. (2023). Burn Evaluation and Management (Archived).

[12] Rastegar Lari A., Alaghehbandan R. (2019). Epidemiological Study of Self-Inflicted Burns in Tehran, Iran. J. Burn Care Rehabil..

[13] Othman N., Kendrick D. (2010). Epidemiology of burn injuries in the East Mediterranean region: A systematic review. BMC Public Health.

[14] Kouchek M., Aghakhani K., Dahmardehei M., Memarian A. (2024). Demographic Assessment of Burn Injuries in Iranian Patients. Bull. Emerg. Trauma.

[15] Mobayen M., Ghaffari M.E., Shahriari F., Gholamrezaie S., Dogahe Z.H., Chakari-Khiavi A. (2021). The Epidemiology and Outcome of Burn Injuries in Iran: A Ten-Year Systematic Review and Meta-Analysis. https://www.researchsquare.com/article/rs-505860/v1.

[16] Kawalec A., Pawlas K. (2020). Home environment and burns in children. Burn. Open.

[17] Latenser B.A. (2007). National Burn Repository 2006: A ten-year review. J. Burn. Care Res..

[18] Postoiu R.L., Onose G. (2022). Research on the possibilities of a therapeutic approach through physical interventions with Laser MLS (Multiwave Locked System) in post-combustion pathology (burns and severe burns). Balneo PRM Res. J..

[19] Salinas J., Chung K.K., Mann E.A., Cancio L.C., Kramer G.C., Serio-Melvin M.L., Renz E.M., Wade C.E., Wolf S.E. (2011). Computerized decision support system improves fluid resuscitation following severe burns: An original study. Crit. Care Med..

[20] Monstrey S. (2008). Burn wound assessment and management. Burns.

[21] Church D., Elsayed S., Reid O., Winston B., Lindsay R. (2006). Burn wound infections. Clin. Microbiol. Rev..

[22] Summer G.J., Puntillo K.A., Miaskowski C., Green P.G., Levine J.D. (2007). Burn injury pain: The continuing challenge. J. Pain.

[23] Hamblin M.R. (2017). Mechanisms and applications of the anti-inflammatory effects of photobiomodulation. AIMS Biophys..

[24] Casella A.J. (1964). Basic Theoretical Considerations of Light Amplification by Stimulated Emission of Radiation (Laser) and An Experimental Study of the Neodymium-Doped Glass Laser. Master’s Thesis.

[25] Kui A., Paraschiv A.M., Pripon M., Chisnoiu A.M., Iacob S., Berar A., Popa F., Gorcea S., Buduru S. (2025). From Pain to Recovery: The Impact of Laser-Assisted Therapy in Dentistry and Cranio-Facial Medicine. Balneo PRM Res. J..

[26] Gordon J.P., Zeiger H.J., Townes C.H. (1954). The maser—New type of microwave amplifier, frequency standard, and spectrometer. Phys. Rev..

[27] Zati A., Valent A. (2008). Laser Therapy in Medicine.

[28] Hamblin M.R., Demidova T.N. (2006). Mechanisms of low level light therapy. Proc. SPIE.

[29] Bjordal J.M., Johnson M.I., Iversen V., Aimbire F., Lopes-Martins R.A.B. (2006). Low-level laser therapy in acute pain: A systematic review of possible mechanisms of action and clinical effects in randomized placebo-controlled trials. Photomed. Laser Surg..

[30] Posten W., Wrone D.A., Dover J.S., Arndt K.A., Silapunt S., Alam M. (2005). Low-level laser therapy for wound healing: Mechanism and evidence. Dermatol. Surg..

[31] Rahmad, Qomarianty A.L., Rachmawati S., Mulia E.R., Purbasari B., Fauzi Y.R., Zainul A., Djaharuddin I., Waluyo Y., Damara V.A. (2025). Effects of Adjunct Low-Level Laser Therapy and Neuromuscular Electrical Stimulation on Pulmonary Rehabilitation in the Intensive Care Unit: A Retrospective Study. Balneo PRM Res. J..

[32] Insero G., Fusi F., Romano G. (2023). The safe use of lasers in biomedicine: Principles of laser-matter interaction. J. Public Health Res..

[33] Baryza M.J., Baryza G.A. (1995). The Vancouver Scar Scale: An administration tool and its interrater reliability. J. Burn. Care Rehabil..

[34] Breivik H., Borchgrevink P.C., Allen S.M., Rosseland L.A., Romoundstad L. (2008). Assessment of pain. Br. J. Anaesth..

[35] Elman S., Hynan L.S., Gabriel V., Mayo M.J. (2010). The 5-D itch scale: A new measure of pruritus. Br. J. Dermatol..

[36] Katz S., Ford A.B., Moskowitz R.W., Jackson B.A., Jaffe M.W. (1963). Studies of illness in the aged: The index of ADL: A standardized measure of biological and psychosocial function. JAMA.

[37] Mahoney F.I., Barthel D.W. (1965). Functional evaluation: The Barthel Index. Md. State Med. J..

[38] Burckhardt C.S., Anderson K.L. (2003). The Quality of Life Scale (QOLS): Reliability, Validity, and Utilization. Health Qual. Life Outcomes.

[39] ASA Laser MLS Laser Therapy (M6)—Research and Therapeutic Solutions. https://www.asalaser.com/en/mlsr-laser-therapy/m6.

[40] ASA Laser MLS in Wound Healing: Research and Therapeutic Solutions. https://www.asalaser.com/.

[41] Chaves M.E., Araújo A.R., Piancastelli A.C., Pinotti M. (2014). Effects of low-power light therapy on wound healing: LASER x LED. An. Bras. Dermatol..

[42] Xiao-Wu W., Herndon D.N., Spies M., Sandord A.P., Wolf S.E. (2002). Effects of Delayed Wound Excision and Grafting in Severely Burned Children. Arch. Surg..

[43] Chipp E., Charles L., Thomas C., Whiting K., Moiemen N., Wilson Y. (2017). A prospective study of time to healing and hypertrophic scarring in paediatric burns: Every day counts. Burn. Trauma.

[44] Postoiu R.L., Marinescu S., Onose G. (2023). Multiwave Locked System LASER photobiomodulation in the multidisciplinary team approach/management of a 3rd degree burn on the posterior thorax in an 82-year-old woman—A case study. Balneo PRM Res. J..

[45] Frozanfar A., Ramezani M., Rahpeyma A., Khajehahmadi S., Arbab H.R. (2013). The effects of low level laser therapy on the expression of collagen type I gene and proliferation of human gingival fibroblasts (Hgf3-Pi 53): In vitro study. Iran. J. Basic Med. Sci..

[46] Ezzati K., Fekrazad R., Raoufi Z. (2019). The effects of photobiomodulation therapy on post-surgical pain. J. Lasers Med. Sci..

[47] Greenhalgh D.G. (2016). Burn Care for General Surgeons and General Practitioners.

[48] Goutos I., Dziewulski P., Richardson P.M. (2009). Pruritus in burns: Review article. J. Burn. Care Res..

[49] Falanga V. (2005). Wound healing and its impairment in the diabetic foot. Lancet.

